# CAMBer: an approach to support comparative analysis of multiple bacterial strains

**DOI:** 10.1186/1471-2164-12-S2-S6

**Published:** 2011-07-27

**Authors:** Michal Wozniak, Limsoon Wong, Jerzy Tiuryn

**Affiliations:** 1 Faculty of Mathematics, Informatics and Mechanics, University of Warsaw, Poland; 2 School of Computing, National University of Singapore, Singapore

## Abstract

**Background:**

There is a large amount of inconsistency in gene structure annotations of bacterial strains. This inconsistency is a frustrating impedance to effective comparative genomic analysis of bacterial strains in promising applications such as gaining insights into bacterial drug resistance.

**Results:**

Here, we propose CAMBer as an approach to support comparative analysis of multiple bacterial strains. CAMBer produces what we called multigene families. Each multigene family reveals genes that are in one-to-one correspondence in the bacterial strains, thereby permitting their annotations to be integrated. We present results of our method applied to three human pathogens: *Escherichia coli*, *Mycobacterium tuberculosis* and *Staphylococcus aureus*.

**Conclusions:**

As a result, more accurate and more comprehensive annotations of the bacterial strains can be produced.

## Background

Large amounts of genomic information are currently being generated, including whole-genome sequences of multiple strains of many bacterial species. The availability of these sequences provides exciting opportunities and applications for comparative genomic analysis of multiple bacterial strains. For example, comparative genomic analysis of the avirulent H37Ra and virulent H37Rv strains of *M. tuberculosis* provides insights into the virulence and pathogenesis of *M. tuberculosis*[1]. As another example, comparative genomic analysis of three linezolid-resistant *S. pneumoniae* strains identified three mutations and the associated genes involved in antibiotic resistance [2]. As a last example, an ingenious comparative genomic analysis of susceptible and resistant strains of *M. tuberculosis* and *M. smegmatis* found that the only gene commonly affected in all three resistant strains encodes atpE, thereby uncovering the mode of action of the novel class of compound Diarylquinoline to be the inhibition of the proton pump of *M. tuberculosis* ATP synthase [3].

These impressive results were achieved by integrating and connecting information generated during the sequencing of multiple distinct strains of the bacteria species mentioned. In order to repeat these past successes, there is a need for a general annotation consensus, as the physical and functional annotations of the strains of the same bacteria differ significantly in some cases. As an extreme case of the problem, the strains of *E. coli* reportedly have only 20% of their genes in common [4]. One cause for the inconsistency of gene annotations is sequencing errors. For example, surprised by the higher similarity between H37Ra and CDC1551 *M. tuberculosis* strains than that between H37Ra and H37Rv, Zheng et al. [1] re-sequenced the relevant loci in H37Rv and discovered a mere 6 out of 85 of the variations were genuine and the rest were sequencing errors [1]. A second cause for gene annotation inconsistency is gene structure prediction errors. For example, when Wakaguri et al. determined the entire sequences of 732 cDNAs in *T. gondii* to evaluate earlier annotated gene models of *T. gondii* at the complete full-length transcript level, they found that 41% of the gene models contained at least one inconsistency [5]. Also, a persistent weakness of gene structure prediction methods is the accuracy of start codon assignment [6], giving rise to a significant amount of gene annotation inconsistency from the resulting gene size variations. Another cause for the inconsistency of gene annotations is the inability to put genes from different strains into correct gene families. For example, the extreme case of *E. coli* is probably due to the simple-minded BLAST reciprocal pairwise comparison that was used in [4] to identify genes belonging to the same gene family. This strategy may identify as few as 15% of genes that are known to have evolutionary relationship; a more sophisticated strategy based on linking by intermediate sequences—a strategy that we also adopt—may increase the ability to recognize genes evolutionary relationship by 70% [7].

This is a frustrating state of affairs for both biologists and bioinformaticians. Therefore, we require structured, exhaustive, comparative databases. While broad-based, web-technology-enabled community annotation has been proposed as a solution to the problem [8], it is feasible only for species having a large interested research community. Unfortunately, this may not be the case for many bacterial strains such as *M. Tuberculosis* due to, for example, insufficient profit opportunity [9]. Another well-known effort is the Fellowship for Interpretation of Genomes [10], which has developed and successfully applied a tool called SEED [11] to support functional annotations of bacterial strains, based on a concept of integrating annotations among multiple bacterial strains in a so-called “subsystem” or gene-family-centric manner. SEED [11] provides functions for navigating and annotating genes such as identifying similar genes from other organisms and comparing their neighborhoods. These functionalities allow users to investigate how a given gene relates to other genes and permit them to update and extend the annotation database via a web interface. However, this process is not automated and the functionalities are more customized for gene function annotation than for gene structure annotation.

Therefore, we should explore the development of alternative approaches and technologies that integrate, connect, and produce consensus gene annotations to support comparative analysis of multiple bacterial strains. We have designed CAMBer to support comparative analysis of multiple bacterial strains. CAMBer approaches the problem as follows. First, we use intermediate sequences—a tactic originally proposed for enhancing FASTA’s ability to detect evolutionary relationship [7]—to link multiple annotations on a gene. We call the resulting structure a *multigene*. Next, multigenes are linked by BLAST edges between their elements into a *consolidation graph*. Multigenes in the same connected component of the graph are proposed to form a family. Finally, we use genomic context information—a tactic originally proposed for enhancing gene function prediction [12]—to refine the consolidation graph. This way we obtain more multigene families where the multigenes in each family are in one-to-one relationship in the bacterial strains considered. These resulting multigene families can be used to support more detailed comparative analysis of multiple bacterial strains for detecting sequencing error, identifying mutations for drug resistance, and other purposes.

In the remainder of this paper, we present the details of CAMBer and our results on *M. tuberculosis*, *S. aureus* and *E. coli*. A preliminary version of CAMBer was described in [13]. The current paper differs from the preliminary version by (i) a more careful analysis and handling of noise due to short possibly erroneous annotations, (ii) testing on more species, (iii) demonstrating scalability on a much larger set of strains, (iv) an analysis of core vs pangenomes, and (v) a substantially revised CAMBer software release—CAMBer is available at http://bioputer.mimuw.edu.pl/camber and can now be run on computer clusters powered by Sun Grid Engine.

## Methods

We present here the details of our approach. We assume that we have a set of bacterial strains whose genomes have been sequenced and annotated. The goal is to arrive at revised annotations of the strains which arise from projecting an annotation of one strain onto the annotations of another. Furthermore, we focus on Translation Initiation Site (TIS) annotations. In this operation, we do not remove the original TIS in the second strain, but rather add new TISs suggested by the annotations of the first one. In particular, we may arrive at new annotated genes in the second strain. In this way, we naturally arrive at the concept of a *multigene* which is just a gene with possibly several TISs.

More precisely: Given an annotation *A* in strain *S*_1_ and let *x* be an ORF which according to *A* encodes a gene in *S*_1_. We run BLAST with query *x* against the sequence of a genome of a strain *S*_2_. Let *y'* be a hit in *S*_2_ returned by BLAST to the query *x* and let *y* be the sequence obtained from *y'* by extending it to the nearest stop codon (in the 3’ direction on the same strand as *y'*)*.* We call *y* an *acceptable hit* with respect to *x* if the following five conditions are satisfied:

• *y* starts with one of the appropriate start codons: ATG, GTG, TTG.

• The BLAST hit *y'* has aligned beginning of the query *x* with the beginning of *y'.*

• The e-value score of the BLAST hit from *x* to *y'* is below a given threshold *e_t_* (typically it is set to 10 ^–10^ or 10 ^–20^).

• The ratio of the length of *y* to *x* is less than 1 + *p_t_* and greater than 1 – *p_t_*, where *p_t_* is a given threshold (typically 0.2 or 0.3). This condition is imposed in order to keep similar lengths of related sequences.

• The percent of identity of the hit (calculated as the number of identities divided by the query length times 100) is above a length-dependent threshold given by the HSSP curve [14]. The curve was originally designed for amino-acid queries, in our case we use the formula:

where *L* is the floor of the number of aligned nucleotide residues divided by 3. Typically *n_t_* is set to 30.5% or 50.5%.

So, assuming that we use BLAST with default parameters, our method has three specific parameters: e-value threshold *e_t_*, length tolerance threshold *p_t_*, and length-depended percent of identity threshold implied by *n_t_.*

It follows from the definition above that an acceptable hit *y* may overlap a gene annotated in *S*_2_ in the same frame, sharing the same stop codon, but having a different TIS. As mentioned above, this gives rise to the notion of a multigene. Different TISs in a multigene *g* give rise to different putative genes. We call them elements of *g.* Obviously genes can be viewed as multigenes with just one element.

Therefore, we have two types of gene structure annotations in the rest of this paper. The first type of annotations are the *original annotations* (of genes) given along with the input sequenced genomes. The second type of annotations are the *predicted annotations* (of multigenes) putatively transferred from one genome to another in the multigene construction and closure processes.

### Consolidation graph

We compute iteratively a closure of annotations which is based on the above described operation of taking acceptable hits. Initially, as step zero, we take original annotations which are furnished with the genomic sequences. Assume that at step *i* ≥ 0 we have an annotation  associated with each strain *S.*

Annotation  in the step *i* + 1 is obtained by taking all acceptable hits in *S* for the queries ranging over all genes annotated in , for every other strain *T.* This process stops when no new acceptable hit is obtained. This process generalizes a proven strategy for identifying more homologs by linking intermediate sequences [7].

**Figure 1:**
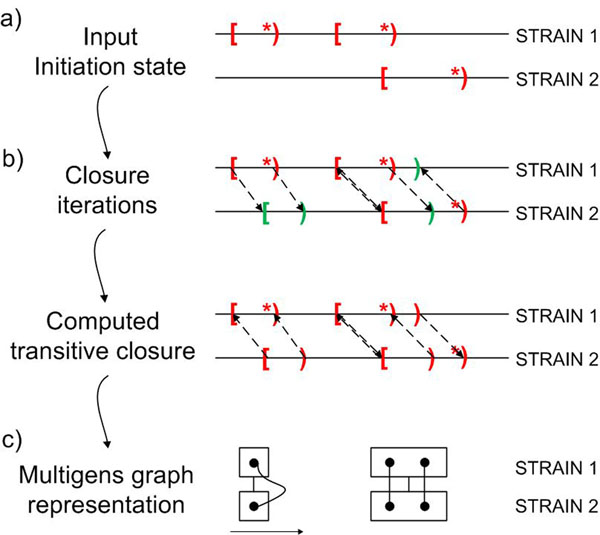
**Schema of the method** Schema of our method to represent the structure of multigenes. For clarity of presentation only one step of the procedure is shown. Square brackets correspond to stop codons of annotated genes, while round brackets with a star correspond to start codons of annotated genes. Round brackets without a star correspond to putative genes indicated by our method (new elements of the multigene). a) Input annotations for strains indicate the initial state of the procedure. b) Dashed arrows indicate acceptable hits. The reader should notice a birth of a second element, rendering a multigene with two elements. c) Two examples of edges in the consolidation graph. Dots represent different elements of a multigene which is represented here as a rectangle. Edges connecting dots represent acceptable hits (we ignore directions here). Edges between rectangles represent edges of the consolidation graph.

Having computed the closure we can construct now a *consolidation graph G.* Its nodes are all multigenes obtained during the process of computing the closure. There is an edge from a multigene *g* to a multigene *g'* if one of the elements of *g'* is obtained as an acceptable hit with respect to one of the elements of *g.* Figure 1 illustrates the process of computing the closure, as well as a construction of the consolidation graph.

### Refinement of the consolidation graph

Connected components of a consolidation graph *G* represent multigene families with a common ancestor. Our next goal is refining the multigene homology relation represented by edges in *G* to obtain as many one-to-one homology classes as possible, i.e. having at most one multigene per strain in such a class. We call a connected component of *G* an *anchor* if it includes at most one multigene for every strain.

One-element anchors are called *orphans. Non-anchors* are the components which fail to be anchors. Obviously the definitions of anchors, orphans, and non-anchors apply to any graph with nodes being multigenes from various strains.

Multigenes in the same anchor are potentially orthologous to each other. In contrast, a non-anchor contains at least two multigenes that are potentially non-orthologous. Genomic context information has been successfully used to clarify gene relationships and improve gene function prediction [12]. So, we propose exploiting genomic context information to analyse and decompose non-anchors into smaller connected subgraphs that can emerge as anchors in the resulting refined consolidation graph.

Our construction of the *refinement* proceeds in stages. At each stage we carry a graph which is a subgraph of the graph from the previous stage. At stage 0, the original consolidation graph *G* is used as the initial input graph *G*^(0)^*.*

**Figure 2:**
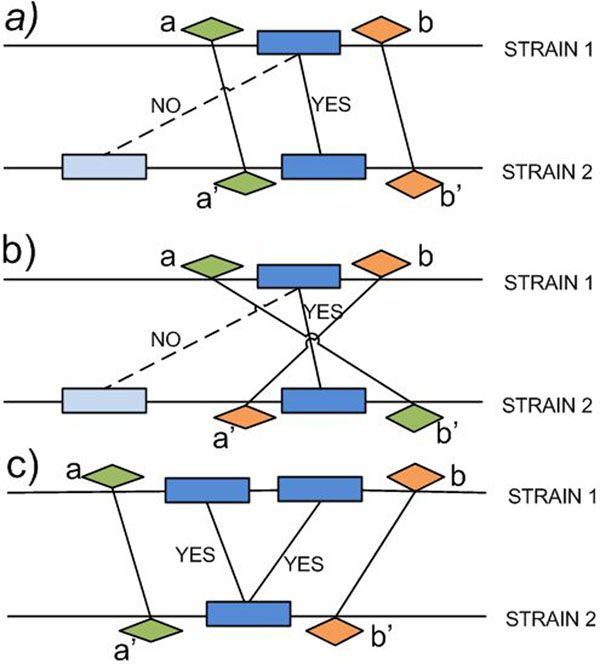
**Refinement procedure** One step of the refinement procedure. Rectangles denote multigenes which belong to non-anchors in the current stage. Rhombus denotes a multigene which is already in an anchor at this stage. Edges connecting rectangles (dashed and solid) are edges of the graph of the current stage. Edges connecting rhombuses are the anchor edges. ‘YES’ means that the edge is keep for the next stage, while ‘NO’ means it is omitted. Parts a) and b) illustrate two situations when we can select one of the edges and leaving out the other. Part c) illustrates the situation when we cannot make such a decision, leading to an unresolved cluster. Both edges are kept in the graph of the next stage. Such a cluster may be resolved at a later stage. Other cases which lead to omitting the edges are possible too.

Suppose we have at stage *i* a graph *G*^(^*^i^*^)^*.* We restrict this graph by performing the following test on each pair (*g*,*g'*) of multigenes originating from strains *S*_1_ and *S*_2_, connected by an edge in *G*^(^*^i^*^)^ which belongs to a non-anchor component of *G*^(^*^i^*^)^*.* Let *a* be the nearest left neighbor multigene of *g* in *S*_1_ which belongs to an anchor of *G*^(^*^i^*^)^ containing a multigene from *S*_2_. Let *b* be the nearest right neighbor multigene of *g* in *S*_1_ which belongs to an anchor of *G*^(^*^i^*^)^ containing a multigene from *S*_2_. In similar way define left (*a'*) and right (*b'*) neighbors of *g'* in *S*_2_. Assuming that all four multigenes *a*, *a'*, *b*, *b'* exist, we keep the edge connecting *g* and *g'* in G^(^*^i^*^+1)^ if either (*a*, *a'*) and (*b*,*b'*) (see Figure 2 (a)), or (*a*, *b*') and (*b*, *a*') (see Figure 2 (b)) are edges in *G*^(^*^i^*^)^, i.e. the corresponding pairs are in the same anchors of *G*^(^*^i^*^)^*.* If at least one of the multigenes *a*, *a'*, *b*, *b'* does not exist, the edge connecting *g* and *g'* in *G*^(^*^i^*^+1)^ is not copied from *G*^(^*^i^*^)^*.* The procedure stops when no edge is removed from the current graph. We call the resulting graph a *refinement* of *G.* Figure 2 (c) shows a situation when we have to retain two edges, leading to a cluster with unresolved one-to-one relationship. These cases may get resolved later when more anchors are obtained.

### Time complexity

The most time consuming operation in the closure procedure is running BLAST. We denote by blast() the BLAST running time. Let *k* be the number of all considered strains and let *n* be the maximal number of annotated genes in the genomes under consideration. For each strain during the closure operation we use every identified or annotated ORF only once. Assuming that the number of newly discovered multigenes does not grow fast, we can estimate the total time of the procedure as *k*^2^ * *n* * blast().

Now, we estimate time complexity of one iteration in the refinement procedure. Again, let *k* be the number of all considered strains and let *n* be the maximal number of identified multigenes among all strains. Denote by *m* the number of non-anchors in the consolidation graph. Additionally, let *p* denote the maximal number of multigenes for one strain among all non-anchor components. In order to find the nearest left and right neighbors of a multigene in linear time we first sort all of them. This takes time *k* ∗ *n* ∗ log *n*. Since we have at most  edges to check for support of the neighboring anchors (checking for support may take time at most *n*), for each of the m non-anchors, it follows that the estimated total time to resolve all of the *m* non-anchors is .

## Results and Discussion

Our approach, called CAMBer, was applied to 9 *M. tuberculosis* (MTB), 22 S. aureus and 41 *E. coli* strains. It was ran with the following parameters: *e_t_* = 10^–10^, *p_t_* = 0.3 and *n_t_* = 30.5%. In our earlier work [13], we used the constant percent of identity threshold (=50%), but finally we decided to use length-dependent percent of identity as we obtained much fewer suspicious very-short predictions. The input datasets comprise nucleotide genome sequences and gene structure annotations of protein-coding genes of the strains in each case study. However, annotations for pseudo genes were filtered. The input datasets were generally taken from GenBank [15], with the exception of six M. tuberculosis strains. The input datasets for three of these strains came from the Broad Institute database; while the remaining three came from the supplementary material of [16].

### Mycobacterium tuberculosis case study

Tuberculosis is still a major cause of deaths worldwide, in particular due to still poorly-understood mechanisms of drug resistance. The first fully sequenced *M. tuberculosis* strain was H37Rv in 1998 and since then several new strains have been sequenced [1,16-18].

**Figure 3:**
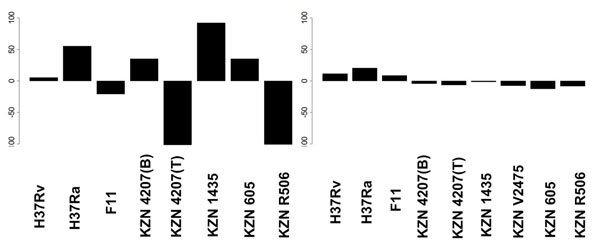
***M. tuberculosis*, before and after the closure** Left: deviation from mean (=3957) in numbers of annotated protein coding genes (KZN V2475 is omitted, because only 101 genes have correct annotation due to a shift in the annotated gene coordinates). Right: deviation from mean (=4146) in numbers of multigenes after unification by the closure procedure. The same scale is used for both charts. Level 0 in the Y axis corresponds to the mean value.

Table 1 gives details of the strains. We notice that there is substantial variance (left box plot in Figure 3) in the number of originally annotated genes. This is probably due to different gene-finding tools and methodologies being applied by different labs, rather than the real genomic composition.

**Table 1:** *M. tuberculosis* dataset

strain ID	source	resist.	# of genes	lab.
H37Rv	NC_000962	DS	3988(26)	S
H37Ra	NC_009525	DS	4034(22)	C
F11	NC_009565	DS	3941(5)	B
KZN 4207(T)	PLoS One. [16]	DS	3902(47)	T
KZN 4207(B)	Broad Institute	DS	3996(4)	B
KZN 1435	Broad Institute	MDR	4059(10)	B
KZN V2475	PLoS One. [16]	MDR	3893(3792)	T
KZN 605	Broad Institute	XDR	4024(26)	B
KZN R506	PLoS One. [16]	XDR	3902(46)	T

Details for input strains for the *M. tuberculosis* case study. The first number in column called *’# of genes’* corresponds to the number of annotated genes, the second (in brackets) corresponds to the number of genes excluded in the study due to unusual start or stop codons or sequence length not divisible by three. In order to avoid ambiguity in naming the same strain sequenced by two labs we introduce an additional suffix (T or B). Characters in last column, called ’*lab.*’, describe the sequencing laboratories: B - The Broad Institute, T - Texas A&M University, C - Chinese National Human Genome Center at Shanghai, S - Sanger Institute.

**Figure 4:**
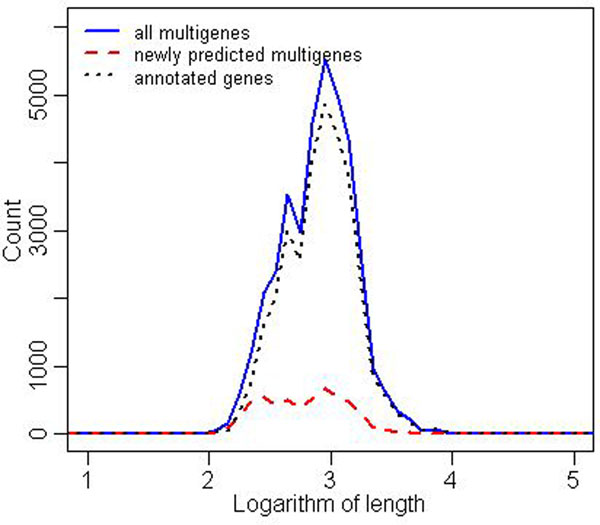
***M. tuberculosis*, distribution of gene lengths** Histograms of gene lengths in logarithmic scale (base = 10) for all *M. tuberculosis* taken together. The x-axis is quantified into ranges of length 0.1. Black dotted line presents the distribution of annotated gene lengths, blue solid line shows the distribution of multigene lengths, red dashed line presents the distribution of length of multigenes with no annotated elements.

It is quite remarkable that variance in the number of predicted multigenes after the closure is much smaller (right box plot in Figure 3). The reader may also compare the corresponding data presented in Tables 1 and 2. Table 2 shows for each strain the distribution of multigenes with respect to the number of elements (TISs). By far the largest group in each strain are one-element multigenes. Also, Figure 4 shows that the predicted multigenes are quite even distributed in terms of gene length.

**Table 2:** Statistics of multigene start sites after the closure procedure for the M. tuberculosis case study. *M. tuberculosis*, multigene start sites statistics Multigene start sites statistics after the closure procedure.

	# of multigenes with					
	5 elt.	4 elt.	3 elt.	2 elt.	1 elt.	total

F11	1	6	68	605	3475	4155
H37Ra	1	5	66	607	3488	4167
H37Rv	1	6	66	602	3483	4158
KZN 605	1	6	68	602	3457	4134
KZN 1435	1	6	69	597	3472	4145
KZN 4207(T)	1	6	70	600	3463	4140
KZN R506	1	6	70	602	3459	4138
KZN V2475	1	6	70	601	3461	4139
KZN 4207(B)	1	5	69	602	3465	4142

The careful reader may have also noticed that the same strain (KZN 4207) sequenced in two labs has quite different numbers of annotated genes (3902 vs. 3996); but after consolidation we have for these two genomes almost the same number of multigenes (4140 vs. 4142).

This case study shows that the method can also be applied to completely unannotated genomes, yielding an initial annotation of a newly sequenced genome. For example, due to a shift in annotation coordinates for the strain KZN V2475 we removed most of the gene annotations (after the shift). Using our method, we were able to annotate 4139 multigenes in the genome.

After refinement of the consolidation graph, the number of connected components rose from 4177 to 4287, but size of the largest component dropped from 127 (there are two such components in the consolidation graph) to 15 (only one such component after refinement). Also the maximal number of multigenes in one strain and in one non-anchor dropped from 17 in the consolidation graph to 3 in the refined consolidation graph.

It is interesting to compare the two largest components of the consolidation graph. As mentioned above they have in total 127 multigenes, each strain having between 12 and 17 multigenes in these non-anchors. What is remarkable here is that H37Rv, having 16 multigenes in each of the two components, has all of these 32 genes annotated in the *Tuberculist* database (http://tuberculist.epfl.ch/) as transposons which belong to the same insertion element (IS6110). Even though these two non-anchors were not successfully resolved by the refinement procedure, the resulting non-anchors (four obtained from each of the original two large non-anchors in the consolidation graph) are pretty small: at most two multigenes per strain.More precisely, each of the original non-anchors was split by the refinement procedure into 34 subclusters (4 non-anchors, and 30 anchors with 9 orphans).

**Figure 5:**
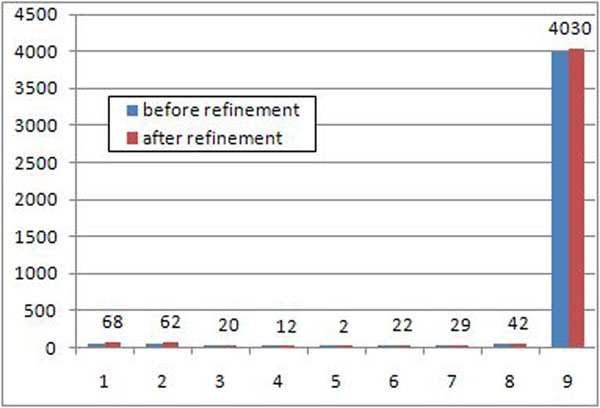
***M. tuberculosis*, distribution of connected components** Histogram of the number of connected components (y-axis) shared by a particular number of strains (x-axis). For better clarity only numbers of connected components after the refinement procedure are shown.

The consolidation graph contains 4177 connected components, with only 43 components (about 1%) being non-anchors and 48 being orphans. After the refinement procedure we obtained slightly more connected components (4287), but the number of non-anchors substantially dropped to 22 (Table 3). Figure 5 gives another point of view for the refinement procedure results.

**Table 3:** Statistics of the connected components before and after refinement for the M. tuberculosis case study. *M. tuberculosis*, before and after refinement Statistics of the connected components before and after refinement.

	# of connected components before refinement	# of connected components after refinement
all connected components	4177	4287

non-anchors	43	22

anchors	4134	4265

orphans	48	68

core anchors	3943	4012

core connected components	3985	4030

With this approach we were also able to discover five cases of gene fusion/fission in the investigated genomes which seems pretty unusual for such closely related strains. We leave the analysis of this phenomenon for further study.

See additional file 1 for detailed summary of the case study results.

### Staphylococcus aureus case study

Since penicillin was introduced for *S. aureus* treatment in 1943, penicillin resistance has become common among *S. aureus* isolates [19]. Two meticillin-resistant strains (N315 and Mu50) are the first fully sequenced *S. aureus* genomes [20].

Genome sequences and annotations of 22 fully sequenced strains were used in our study. At the time of writing, these were the only available S. aureus strains with “completed” sequencing status. Table 4 presents details of the strains.

**Table 4:** S. aureus dataset. Details for input strains for the S. aureus case study. The first number in column called ’# of genes’ corresponds to the number of annotated genes, the second (in brackets) corresponds to the number of genes excluded in the study due to unusual start or stop codons or sequence length not divisible by three.

strain ID	source (GenBank ID)	# of genes	genome length	lab.
TW20 0582	FN433596	2769(5)	3043210	Welcome Trust Sanger Institute
JKD6008	CP002120	2680(0)	2924344	Monash University
JH9	CP000703	2769(5)	2906700	US DOE Joint Genome Institute
JH1	CP000736	2680(0)	2906507	US DOE Joint Genome Institute
MRSA252	BX571856	2697(0)	2902619	Sanger Institute
Mu3	AP009324	2746(0)	2880168	Juntendo University
Newman	AP009351	2655(5)	2878897	Juntendo University
Mu50	BA000017	2699(63)	2878529	Juntendo University
USA300 TCH1516	CP000730	2624(0)	2872915	Baylor College of Medicine
USA300 FPR3757	CP000255	2699(61)	2872769	University of California, San Francisco
ST398 S0385	AM990992	2657(0)	2872582	University Medical Centre Utrecht
ED133	CP001996	2560(0)	2832478	University of Edinburgh
ED98	CP001781	2699(0)	2824404	University of Edinburgh
04-02981	CP001844	2653(2)	2821452	Robert Koch Institute
NCTC 8325	CP000253	2661(0)	2821361	University of Oklahoma Health Sciences Center
MW2	BA000033	2650(59)	2820462	NITE
N315	BA000018	2892(0)	2814816	Juntendo University
JKD6159	CP002114	2632(6)	2811435	University of Melbourne
COL	CP000046	2593(59)	2809422	TIGR
TCH60	CP002110	2555(1)	2802675	Baylor College of Medicine
MSSA476	BX571857	2672(1)	2799802	Sanger Institute
RF122	AJ938182	2673(0)	2742531	University of Minnesota

**Figure 6:**
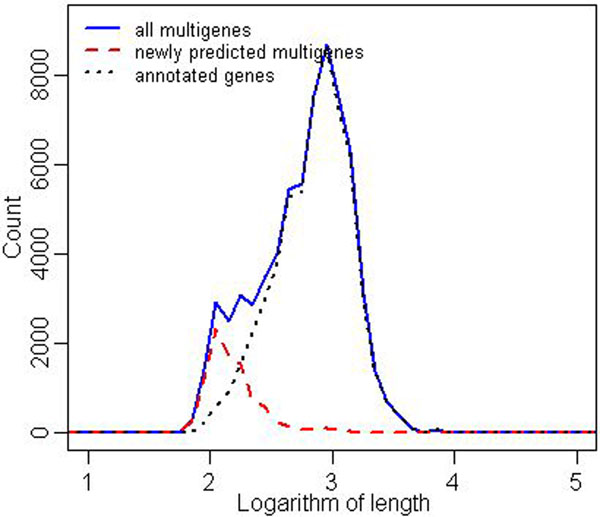
***S. aureus*, distribution of gene lengths** Histograms of gene lengths in logarithmic scale (base = 10) for all *S. aureus* strains taken together. The x-axis is quantified into ranges of length 0.1. Black dotted line presents the distribution of annotated gene lengths, blue solid line shows the distribution of multigene lengths, red dashed line presents the distribution of length of multigenes with no annotated elements.

**Figure 7:**
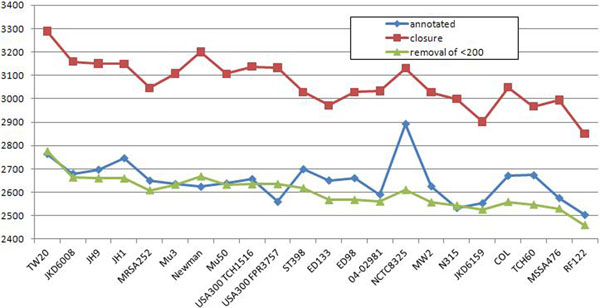
**S. aureus, before and after the closure** This plot presents numbers of annotated genes and numbers of the multigenes after the closure procedure applied to *S. aureus* strains. On the x-axis strains are listed (from left to right) in descending order of their genome length. The blue line *annotated* and the red line *closure* present respectively the number of annotated genes and the number of multigenes (after the closure) for each strain. The green line *removal of* <*200* presents the number of multigenes after the closure and after applied post-processing of removal multigenes shorter than 150 nucleotides length.

In this medium-size case study most of the results and corollaries are similar to the *M. tuberculosis* case study. However, we highlight below three interesting observations. The first observation is that there is a much large number of short predicted multigenes compared to the number of short original annotated genes, as shown in Figure 6. This contrasts sharply with the situation for *M. tuberculosis* depicted in Figure 4. This means that in *S. aureus*, many strains have short original annotations that are annotated to one of them but not to other strains, even though highly homologous regions exist in other strains. This suggests possible higher occurrence of annotation errors in short genes of *S. aureus*, especially in strains like NCTC8325; see Figure 7. The second observation is that the computing of the closure took 8 iterations, which is similar to the much larger study of *E. coli* (8 iterations) and more than the *M. tuberculosis* case study (3 iterations). The third observation is that the maximal number of TISs in a multigene is 13 (see Table 5 for more details), where for *E. coli* it is 9 and for *M. tuberculosis* 5. As in the other case studies we observe uneven distribution in the number of original annotated genes; see Figure 7. To assess the degree of unevenness we calculated the mean absolute difference in counts coming from two neighboring strains, where strains are ordered in decreasing order of the size of their genomes. It is 78 for the original annotation curve vs. 70 for the curve constructed after the closure operation, which further drops to 29 after post-processing removal of multigenes shorter than 200 nucleotides. This inconsistency was probably caused by different gene-finding methodologies applied by different labs. Curves like those presented in Figure 7 allow us also to estimate which labs were more conservative and which were more liberal when calling a given ORF a gene. For example, we observe a big peak in the number of original annotated genes for the strain NCTC8325, suggesting that this is perhaps the case of a more liberal annotation. Indeed, we investigated the number of connected components with multigenes present in all strains but have original annotations in only one strain. It turned out that there are only 7 strains that contribute at least one such connected component, of which the strain NCTC8325 contributes the highest number (22), with the second strain being USA300 TCH1516 (18). All other strains contributed less than 4 such components. An example of a strain with a rather conservative annotation is USA300 FPR3757, as can be clearly seen from a dip of the curve in Figure 7.

**Table 5:** Statistics of multigene start sites after the closure procedure for the S. aureus case study. *S. aureus*, multigene start sites statistics Multigene start sites statistics after the closure procedure.

	# of multigenes with											
	13 elt.	10 elt.	9 elt.	8 elt.	7 elt.	6 elt.	5 elt.	4 elt.	3 elt.	2 elt.	1 elt.	total

TW20	0	0	1	0	1	3	9	45	224	823	2183	3289
JKD6008	0	0	1	0	1	2	8	44	218	827	2058	3159
JH9	0	0	0	1	0	0	10	42	240	805	2052	3150
												
MRSA252	9	0	0	0	1	2	8	44	207	805	1970	3046
Mu3	0	0	0	1	0	0	12	39	235	789	2032	3108
Newman	0	0	1	0	1	2	12	46	231	818	2089	3200
Mu50	0	0	0	1	0	0	12	39	234	788	2033	3107
USA300 TCH1516	0	0	1	0	1	2	12	49	237	815	2020	3137
USA300 FPR3757	0	0	1	0	1	2	12	49	239	813	2016	3133
ST398	0	0	1	0	0	0	6	39	198	768	2017	3029
ED133	0	0	1	0	0	1	9	41	212	762	1946	2972
ED98	0	0	0	1	0	0	11	38	235	769	1974	3028
04-02981	0	0	0	1	0	0	11	40	236	778	1967	3033
NCTC8325	0	0	1	0	1	2	11	44	228	799	2044	3130
MW2	0	0	0	0	0	3	11	45	230	790	1948	3027
N315	0	0	0	1	0	0	12	40	234	765	1947	2999
JKD6159	6	1	0	0	0	0	9	38	208	760	1880	2902
COL	0	0	1	0	1	2	13	49	234	785	1964	3049
TCH60	4	1	1	0	0	1	8	48	192	776	1936	2967
MSSA476	0	0	0	0	0	3	11	42	225	780	1933	2994
RF122	0	0	0	0	0	5	8	40	186	706	1905	2850

It is rather expected that most of the inconsistencies concern short genes, leading to a sudden increase in the number of short multigenes after the closure procedure; see Figure 6. Therefore, it is interesting to investigate the cases where new long multigenes are predicted after the closure. There are in total 31 connected components with multigenes of length at least 300 nucleotides which were originally annotated in less than half of the strains. Two of them have multigenes in all 22 strains with only one originally annotated element. More precisely these two connected components were contributed by genes *SAOUHSC_00630* and *SAOUHSC_01489* annotated in NCTC8325. Both these genes are overlapped by genes which have original annotations in all remaining strains, which suggests that these two genes were perhaps incorrectly annotated.

We also checked the structure of annotations for highly overlapping multigenes as another source of possible inconsistencies in genome annotations. For each strain we searched for pairs of highly overlapping multigenes (after the closure) belonging to core anchors (i.e., anchors with elements in every strain). Here, we define a pair of multigenes as highly overlapping when the length of the overlap is at least 50% of the length of the shorter multigene in the pair. The number of identified pairs of multigenes in one strain varies from 17 to 20, depending on the strain. As it can be expected, strains with more liberal annotations have higher number of annotated overlapping multigene pairs. In particular, NCTC8325 has 7 pairs of multigenes where both multigenes in the pair have at least one original annotated element; ST398 has 5 such pairs; and ED98 has 4. On the other hand, RF122, USA300 FPR3757, Newman, N315 and 8 other strains do not have any such highly overlapping pair of annotated multigenes.

**Figure 8:**
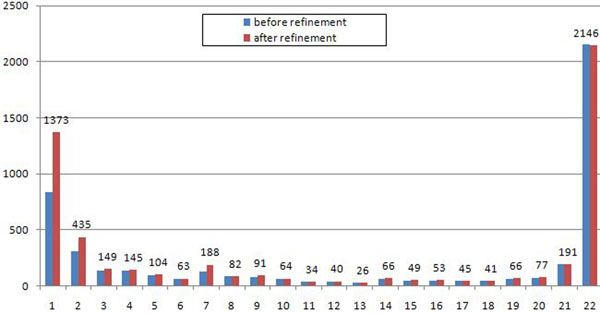
**S. aureus, distribution of connected components** Histogram of the number of connected components (y-axis) shared by a particular number of strains (x-axis).

Table 6 presents statistics of the refinement procedure. After the closure procedure we obtained 273 (around 5%) non-anchors in the consolidation graph, of which the refinement procedure split 210 and completely resolving 175 of them. The refinement procedure yielded 4 new anchors with multigenes in all strains. Figure 8 gives another perspective on the refinement procedure results. See additional file 2 for detailed summary of the case study results.

**Table 6:** Statistics of the connected components before and after refinement for the S. aureus case study. *S. aureus*, before and after refinement Statistics of the connected components before and after refinement.

	# of connected components before refinement	# of connected components after refinement
all connected components	4737	5528

non-anchors	273	107

anchors	4464	5421

orphans	839	1373

core anchors	2115	2119

core connected components	2156	2146

### Escherichia coli case study

*Escherichia coli* is the most well-studied prokaryotic organism and has been used in numerous research studies as a model organism. The strain K-12 MG1655 became the first fully sequenced *E. coli* genome in 1997 [21].

We perform the analysis on *E. coli* to test scalability of CAMBer and check stability of the results on a large dataset. In our case study, we use genome sequences and annotations of 41 fully sequenced strains deposited in NCBI. At the time of writing, these were the only available *E. coli* strains with “completed” status. Table 7 presents details of the strains.

**Table 7:** *E. coli* dataset

strain ID	source (GenBank ID)	# of genes	genome length	lab.
O26:H11 11368	AP010953	5363(4)	5697240	University of Tokyo
O157:H7 EC4115	CP001164	5315(0)	5572075	J. Craig Venter Institute
O157:H7 EDL933	AE005174	5348(10)	5528445	University of Wisconsin
O157:H7 TW14359	CP001368	5263(6)	5528136	University of Washington
O157:H7 Sakai	BA000007	5360(5)	5498450	GIRC
O103:H2 12009	AP010958	5053(4)	5449314	University of Tokyo
O55:H7 CB9615	CP001846	5014(0)	5386352	Nankai University
O111:H 11128	AP010960	4971(4)	5371077	University of Tokyo
042	FN554766	4792(18)	5241977	Welcome Trust Sanger Institute
CFT073	AE014075	5378(4)	5231428	University of Wisconsin
ED1a	CU928162	4914(4)	5209548	Genoscope
UMN026	CU928163	4825(4)	5202090	Genoscope
55989	CU928145	4762(4)	5154862	Institute Pasteur and Genoscope
ETEC H10407	FN649414	4696(3)	5153435	Welcome Trust Sanger Institute
IAI39	CU928164	4731(7)	5132068	Genoscope
ABU 83972	CP001671	4793(6)	5131397	Georg-August-University Goettingen
IHE3034	CP001969	4757(3)	5108383	IGS
APEC O1	CP000468	4467(3)	5082025	Iowa State University
SMS-3-5	CP000970	4742(3)	5068389	TIGR
UTI89	CP000243	5066(13)	5065741	Washington University
S88	CU928161	4695(3)	5032268	Genoscope
UM146	CP002167	4650(0)	4993013	MBRI
E24377A	CP000800	4755(0)	4979619	TIGR
O127:H6 E2348/69	FM180568	4553(4)	4965553	Sanger Institute
536	CP000247	4629(2)	4938920	University of Goettingen
W	CP002185	4478(4)	4900968	AIBN/KRIBB
SE11	AP009240	4679(0)	4887515	Kitasato Institute for Life Sciences
O83:H1 NRG 857C	CP001855	4429(13)	4747819	Public Health Agency of Canada Laboratory for Foodborne Zoonoses
ATCC 8739	CP000946	4180(7)	4746218	US DOE Joint Genome Institute
SE15	AP009378	4338(0)	4717338	Kitasato University
IAI1	CU928160	4353(4)	4700560	Genoscope
K-12 substr. DH10B	CP000948	4125(5)	4686137	University of Wisconsin-Madison
K-12 substr. W3110	AP009048	4225(9)	4646332	Nara Institute of Science and Technology
HS	CP000802	4383(3)	4643538	TIGR
K-12 substr. MG1655	U00096	4144(7)	4639675	University of Wisconsin-Madison
DH1	CP001637	4159(4)	4630707	US DOE Joint Genome Institute
BL21-Gold(DE3)pLysS	CP001665	4208(8)	4629812	US DOE Joint Genome Institute
BW2952	CP001396	4083(5)	4578159	TEDA School of Biological Sciences and Biotechnology
BL21(DE3) BL21	AM946981	4227(4)	4570938	Austrian Center for Biopharmaceutical Technology
B REL606	CP000819	4158(6)	4558953	International *E. coli* B Consortium
BL21(DE3)	CP001509	4181(23)	4558947	Korea Research Institute of Bioscience and Biotechnology

Details for input strains for the *E. coli* case study. The first number in column called ’# of genes’ corresponds to the number of annotated genes, the second (in brackets) corresponds to the number of genes excluded in the study due to unusual start or stop codons or sequence length not divisible by three.

Figure 8 presents a distribution of gene (original annotation) and multigene (after applying our closure procedure) counts for the 41 strains. Strains in this plot occur (from left to right) in decreasing order of sizes of their genomes. We observe that the curve based on the original annotations is quite bumpy, which reflects incongruence of annotations made by different labs. This observation is supported by computing an average absolute difference in counts coming from two neighboring strains: it is 152.1 for the original annotation curve vs. 95.6 for the curve constructed after the closure operation; and it is only 64 after post-processing removal of multigenes shorter than 200 nucleotides was applied.

**Figure 9:**
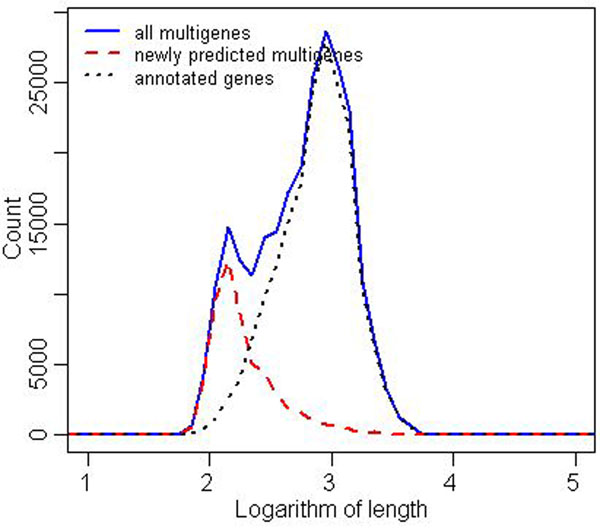
***E. coli*, distribution of gene lengths** Histograms of gene lengths in logarithmic scale (base = 10) for all *E. coli* strains taken together. The x-axis is quantified into ranges of length 0.1. Black dotted line presents the distribution of annotated gene lengths, blue solid line shows the distribution of multigene lengths, red dashed line presents the distribution of length of multigenes with no annotated elements.

**Figure 10:**
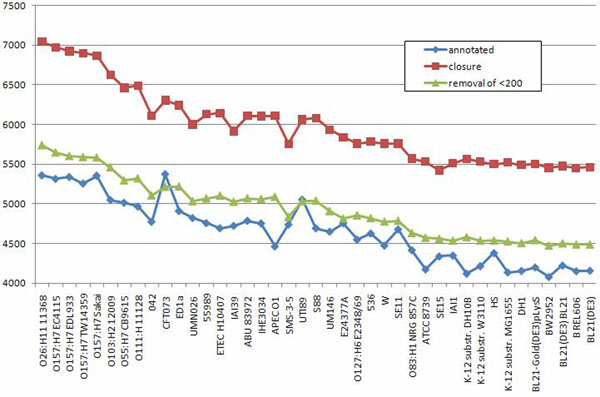
***E. coli*, before and after the closure** This plot presents numbers of annotated genes and numbers of the multigenes after the closure procedure applied to E. coli strains. On the x-axis strains are listed (from left to right) in descending order of their genome length. The blue line annotated and the red line closure present respectively the number of annotated genes and the number of multigenes (after the closure) for each strain. The green line removal of < 200 presents the number of multigenes after the closure and after applied post-processing removal of multigenes shorter than 200 nucleotides length.

We have also analyzed the distribution of sizes of the newly predicted multigenes. Figure 9 presents these distributions for all *E. coli* strains taken together. The striking feature is that most of the newly predicted multigenes are pretty short, around 200 nucleotides. Of course each such newly predicted multigene must have a witness coming from an original annotation in another strain. This distribution suggests that annotations of short genes may be a possible source of annotation errors. It also suggests one should remove very short multigenes from global considerations. The distribution after removal is flatter, resembling closer to the distribution for original annotated genes, as shown in Figure 10.

It is also interesting to investigate which strains had the most liberal annotations of genes. This can be seen by considering connected components which have an element in each strain, but only one gene in such a component has original annotation. Such a situation suggests that the lab which was annotating this strain annotated the ORF as a gene, while other labs did not, even though the corresponding ORF was present in genomes that the other labs were working on. The top 5 most liberal annotations were obtained for CFT073 (37 components), E24377A (22 components), O157-H7 EC4115 (13 components), UTI89 (12 components), and IAI1 (10 components). For the rest of the strains, the number of such components was smaller than 8. In total, there were 22 strains of *E. coli* which contributed components described above. Adopting a similar approach as in the *S. aureus* case study we performed the analysis of annotations for highly overlapping multigenes viewed as another source of inconsistencies in genome annotations. In the case of *E. coli* strains, the number of highly overlapping pairs of multigenes varies in strains from 167 to 172.

Again, strains with local maxima on the curve of annotated genes (see Figure 10) tend to have a higher number of pairs of highly overlapping multigenes with both multigenes annotated. In particular, CFT073 has 86, UTI89 has 76, and E24377A has 30. On the other hand, APECO1 has only one such pair.

Even though there are known cases of functional genes with untypical start codons, we decided to restrict our attention to the three typical start codons (ATG, GTG, CTG), hoping that it does not influence our results in a substantial way. However, it is interesting to follow the fate of genes which have untypical start codons in some strains. For example, the first fully sequenced *E. coli* strain (K-12 MG1655) has annotated two protein-coding genes with untypical start codons. The first gene is *infC*, encoding IF3 translation initiation factor. As discussed in [22], this untypical start codon (ATT) may be in use for self-regulation. Interestingly, using CAMBer, we revealed that annotations for 25 (i.e., more than half) of the studied strains have annotated a shorter version of the gene (435 nucleotides instead of 543) with the GTG start codon. The second gene, *htgA* (synonym *htpY* ), is involved in heat shock response. The possible explanation for the untypical start codon (CTG) was discussed in [23]. Using CAMBer, we identified 7 strains which annotated this gene with a different TIS. Six of them have annotated 495 nucleotides as gene length and one 486. In both cases, GTG was selected as the start codon. It is possible that some other start codons may also be used in *E. coli*[21].

**Figure 11:**
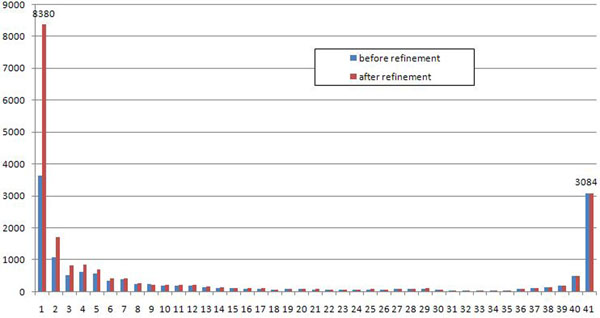
***E.coli*, distribution of connected components** Histogram of the number of connected components (y-axis) shared by a particular number of strains (x-axis).

In this case study the maximal number of TIS in a multigene is 9; see Table 8 for more details. Interestingly, it is less than for *S. aureus* — the medium-size case study; see Table 5. Table 9 presents statistics of the refinement procedure. After the closure procedure we obtained 1176 non-anchors, of which we were able to split 934 using the refinement procedure, 689 of them we resolved completely into anchors. The refinement procedure produced only two new anchors with multigenes in all strains. Most of the connected components obtained were small, in particular, the number of orphans doubled; see Figure 11.

**Table 8:** Statistics of multigene start sites after the closure procedure for the E. coli case study. *E. coli*, multigene start sites statistics Multigene start sites statistics after the closure procedure.

	# of multigenes with									
	9 elt.	8 elt.	7 elt.	6 elt.	5 elt.	4 elt.	3 elt.	2 elt.	1 elt.	total

O26-H11-11368	7	7	4	20	57	213	631	1793	4310	7042
O157-H7-EC4115	13	13	7	14	62	157	624	1857	4226	6973
O157-H7-EDL933	10	13	5	18	46	143	617	1831	4242	6925
O157-H7-TW14359	14	12	7	13	58	151	616	1836	4195	6902
O157-H7-Sakai	14	8	5	16	49	152	600	1826	4198	6868
O103-H2-12009	0	28	3	16	53	162	583	1704	4078	6627
O55-H7-CB9615	0	4	10	12	44	156	564	1722	3950	6462
O111-H-11128	35	7	1	18	54	154	565	1686	3970	6490
042	0	2	2	11	28	138	538	1598	3791	6108
CFT073	6	2	4	8	33	161	534	1721	3836	6305
ED1a	1	4	0	11	24	144	524	1577	3957	6242
UMN026	0	3	7	9	29	139	539	1556	3719	6001
55989	0	2	3	11	37	146	559	1605	3766	6129
ETECH10407	1	3	2	11	36	143	549	1589	3809	6143
IAI39	22	5	2	4	43	149	508	1619	3566	5918
ABU83972	0	3	3	7	29	140	530	1662	3736	6110
IHE3034	0	1	2	9	32	144	563	1644	3712	6107
APECO1	0	1	2	12	29	145	542	1675	3705	6111
SMS-3-5	3	0	5	8	24	116	500	1515	3586	5757
UTI89	1	1	2	9	30	147	561	1655	3658	6064
S88	0	2	3	9	33	149	550	1658	3678	6082
UM146	1	1	1	8	28	137	528	1590	3640	5934
E24377A	0	1	2	6	31	125	516	1502	3656	5839
O127-H6-E234869	0	3	2	8	15	169	471	1474	3618	5760
536	1	0	2	8	21	135	510	1560	3546	5783
W	0	1	2	6	27	112	483	1492	3636	5759
SE11	0	3	0	9	32	119	505	1467	3625	5760
O83-H1-NRG857C	0	1	2	7	23	117	489	1503	3427	5569
ATCC8739	0	1	3	6	26	106	491	1431	3468	5532
SE15	0	1	1	10	22	111	467	1445	3366	5423
IAI1	0	1	1	5	29	121	484	1442	3428	5511
K12-DH10B	0	3	1	6	23	98	457	1475	3504	5567
K12-W3110	0	3	1	6	25	100	458	1467	3471	5531
HS	0	0	1	7	24	121	480	1439	3429	5501
K12-MG1655	0	3	1	6	25	97	463	1455	3473	5523
DH1	0	3	1	6	25	97	458	1453	3447	5490
B-REL606	0	3	2	5	24	99	511	1472	3389	5505
BW2952	0	3	1	7	25	97	453	1447	3421	5454
BL21-Gold-DE3	0	2	1	5	25	98	497	1460	3388	5476
BL21-DE3	0	2	1	5	25	100	497	1461	3362	5453
BL21-DE3-BL21	0	2	1	5	25	100	497	1461	3370	5461

**Table 9:** Statistics of the connected components before and after refinement for the E. coli case study. *E. coli*, before and after refinement Statistics of the connected components before and after refinement.

	# of connected components before refinement	# of connected components after refinement
all connected components	13973	20257

non-anchors	1176	563

anchors	12797	19694

orphans	3637	8380

core anchors	2963	2979

core connected components	3089	3084

See additional file 3 for detailed summary of the case study results.

### Core genome vs. pangenome

Finally, we computed core genome and pangenome for the family of *E. coli* strains using our concept of a multigene and compared the result to the core genome and pangenome computed along the lines described in [4]. The latter paper considered 61 strains, many of them not having the sequencing status of “completed”. Our set of strains is not a subset of the 61 strains mentioned above since there were some newly published strains (e.g., *E. coli* UM146, published in January 2011). For this reason, we had to repeat the computations as described in [4] for our set of strains.

As in [4] we call two genes homologous if the percent of identity is at least 50% covering at least 50% of the longer gene. We order all strains with respect to decreasing size of their genomes. We start with the strain having the largest genome, initializing both the pangenome and the core genome equal to the set of all genes of that strain. In the *n*-th step, we put a gene of the *n*-th strain into the pangenome if it is not homologous to any of the genes of the previously considered strains. We also remove a gene from the core genome when it not homologous to any of the genes of the *n*-th strain.

**Figure 12:**
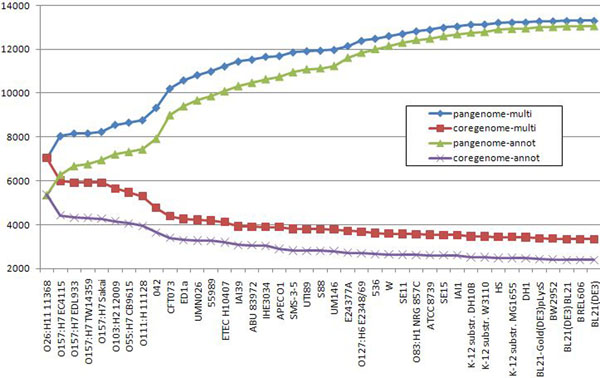
***E. coli*, core genome vs pangenome** Core vs. pangenome plots of 41 *E. coli* strains calculated using original annotations and multigene annotations, predicted by CAMBer. Strains are sorted (from left to right) in descending order of their genome sizes. Violet and green (*coregenome-annot* and *pangenome-annot*) lines connect cumulative numbers of core and pangenome sizes using annotated genes, while red and blue (*coregenome-multi* and *pangenome-multi*) lines connect cumulative numbers of core and pangenome sizes using multigenes after the closure procedure. The proportion of core genome to pangenome size has risen from 18% to 25% after the closure.

We run two experiments on our set of strains: one which relies on the original gene annotations, as it was done in [4], and another one which relies on previously pre-computed multigenes. Figure 12 shows the dynamics of change in gene numbers both for pangenome and core genome. It shows that as the number of strains increases both methods asymptotically converge to a pangenome size of around 13 000 genes. This suggests that the notion of a pangenome is quite robust when considering a large number of strains. On the other hand, there is a consistent difference between sizes of the core genome computed for the original annotations vs. pre-computed multigenes. For the latter method the core genome is substantially larger than for the former, resulting in an increase of the percentage with respect to pangenome from 18% to 25%. The analogous percentage for the 61 strains considered in [4] was reported in that paper as only 6%, but the computation was relying on original annotations.

**Figure 13:**
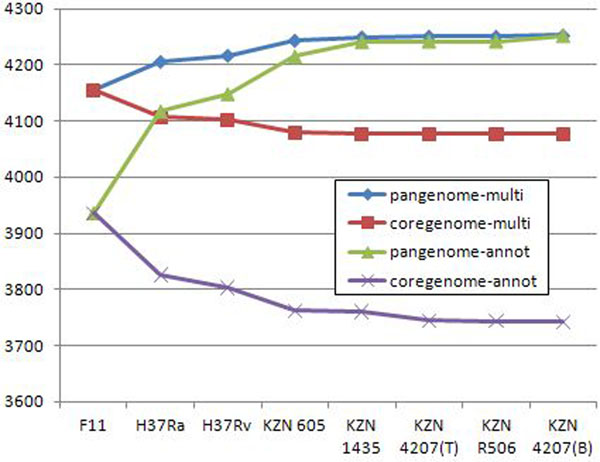
***M. tuberculosis*, core genome vs pangenome** Core vs. pangenome plots of 9 *M. tuberculosis* strains calculated using original annotations and multigene annotations, predicted by CAMBer. Strains are sorted (from left to right) in descending order of their genome sizes. Violet and green (*coregenome-annot* and *pangenome-annot*) lines connect cumulative numbers of core and pangenome sizes using annotated genes, while red and blue (*coregenome-multi* and *pangenome-multi*) lines connect cumulative numbers of core and pangenome sizes using multigenes after the closure procedure. The strain KZN V2475 was excluded due to wrong annotation, caused by a shift in gene coordinates. The proportion of core genome to pangenome size has risen from 88.5% to 96.1% after the closure.

**Figure 14:**
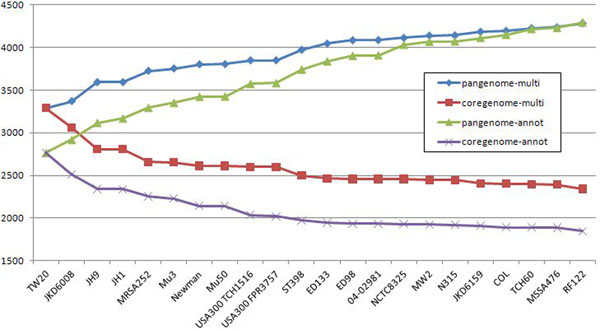
**S. aureus, core genome vs pangenome** Core vs. pangenome plots of 22 *S. aureus* strains calculated using original annotations and multigene annotations, predicted by CAMBer. Strains are sorted (from left to right) in descending order of their genome sizes. Violet and green (*coregenome-annot* and *pangenome-annot*) lines connect cumulative numbers of core and pangenome sizes using annotated genes, while red and blue (*coregenome-multi* and *pangenome-multi*) lines connect cumulative numbers of core and pangenome sizes using multigenes after the closure procedure. The proportion of core genome to pangenome size has risen from 42% to 52% after the closure.

We also performed a similar analysis on *M. tuberculosis* and *S. aureus* strain families. Figures 13 and 14 present results for *M. tuberculosis* and *S. aureus* respectively. The conclusions are similar as for *E. coli*. The size of pangenome computed using both methods converges, as the number of considered strains increases. On the other hand size of the core genome shows a consistent difference for both methods. As a result the proportion of the size of core genome with respect to the pangenome substantially depends on the chosen method, yielding higher score for the method based on pre-computed multigenes. The increase is from 42% to 52% for *S. aureus* and from 88% to 96% for *M. tuberculosis*.

## Conclusions

As the number of sequenced genomes of closely related bacterial strains grows, there is a need to join and consolidate different annotations of genomes. It turns out that annotations of related strains are often inconsistent in declaring Translation Initiation Sites (TIS) for the corresponding homologous genes. They also sometimes miss a gene in a segment which sequence-wise is very similar to a segment in the genome of another species which is declared as a gene. We propose in this paper a methodology which consists in collecting all possible different TISs, as well as genes which are present sequence-wise in a strain but whose annotation is missing. We believe this is the right approach toward correcting annotations.

To achieve this goal we constructed a *consolidation graph* which is based on the concept called here a *multigene*. Multigene is an entity which combines all different TISs derived from sequence comparisons with genes annotated in other strains, or genes which were already established as multigenes. The transitive closure of this operation on all genomes of interest gives the space of multigenes. Multigenes serve as nodes of the consolidation graph. Each TIS in a multigene gives rise to a gene which we called an *element* of the multigene. All elements of a given multigene share the same stop codon. Each multigene with more than one element can be viewed as a task of deciding on the right TIS. Such a decision may have to involve some wet lab experiments or consideration of ESTs or 5’ cDNAs [5]. This issue is not discussed in the present paper. So conceptually a multigene corresponds to a gene in which a TIS is yet to be determined (hopefully by selecting one of the listed start sites).

Why does genome alignment not give similar results as the consolidation graph? The main reason is that in genome alignment one works with sequences which are fragments of genomes without paying any attention to functional genetic elements. In this way one discovers genomic areas of high similarity. Even though postprocessing is often performed, by considering functional genomic elements and the homology relationship between genes or revised genes, gene annotation is not always correctly reconstructed. Moreover, pairwise genome alignment approaches may also miss homologous fragments that can only be linked by intermediate sequences [7]. In contrast, in the consolidation graph we start with annotated genes and close up iteratively with the sequences which come out as significant BLAST hits to the queries already obtained in this analysis. There is a caveat to this iteration process however. In particular, when the input contains a conserved genomic region that is incorrectly annotated as a gene in one strain, CAMBer may fish out homologous regions from other strains and propagate the incorrect gene structure annotation to them. Connected components of the consolidation graph naturally define sets of multigenes which might be called *multigene families*. This concept of a multigene family is rather new, since in the multigene family construction we did not rely exclusively on given annotations. It turns out that these multigene families can be used to reconstruct a *one-to-one homology* relation for most of the genes. This procedure we call *refinement*. For this we start off with families which consist of at most one multigene from each strain. These we called *anchors*. Then we extend the one-to-one homology relation by considering a genome position of genes, which were not yet related by the one-to-one relationship, with respect to the anchors. This method leaves unresolved only very few small families which presumably should be further curated manually. The one-to-one relationship can be used, among other things, in deciding which multiple alignments should be considered for detection of possible mutations, or even detection of possible sequencing errors.

The methodology above was illustrated with three case studies on 9 *Mycobacterium tuberculosis*, 22 *Staphylococcus aureus* and 41 *Escherichia coli* strains. It is evident from the results presented in this paper that genome annotations done in different labs were not congruent to each other. After performing the consolidation, variance in the total gene count is much smaller than before, suggesting that the revised annotations could lead to a more coherent view of functional elements in various strains.

Analyzing CAMBer results, we find out that most of the inconsistencies are related to short genes. Moreover, we find huge disagreement in annotations of highly overlapping ORF’s, located in different reading frames (possibly on the opposite strand). Comparing annotations of pairs of highly overlapping multigenes belonging to core anchors, we found many inconsistencies in them. For example, the S. aureus strain NCTC8325 has originally annotated both highly overlapping multigenes in 7 such pairs, whereas 10 out of 22 strains have no such a pair at all. This observation suggests that an analysis of overlapping genes should use annotations with caution. The issue of possibly missing annotations in the case of overlapping genes was previously mentioned in [24].

The *M. tuberculosis* case study showed that CAMBer can also be applied to completely unannotated genomes, yielding an initial annotation of a newly sequenced genome. This case may be illustrated with strain KZN V2475. Presumably due to a shift in annotation coordinates most genes of this strain have clearly incorrect annotations (see the corresponding entry in Table 1). For this reason we have discarded the originally published annotation for this strain and run CAMBer on the remaining annotated strains plus unannotated KZN V2475. As can be seen in Table 2 we were able to retrieve annotations for KZN V2475 which look quite similar in terms of statistics as annotations for the other strains.

We computed the core genome and pangenome for *M. tuberculosis*, *S. aureus* and *E. coli* strain families using two approaches: one that relies on originally annotated genes (along the lines of [4]) and another which uses our notion of a multigene. Interestingly, both methods give similar results for pangenome, but they significantly differ on the core genome, with the latter method producing larger result. Both these observations hold true for all three case studies. The proportion of the core genome size to the pangenome size increases from 18% to 25% for *E. coli*, from 42% to 52%; for *S. aureus*, and from 88% to 96% for *M. tuberculosis*, when switching from the former to the latter method. We suggest that the method based on pre-computed multigenes, as it is done by CAMBer, gives a more reliable estimate of the core genome. However, it is probably the case that the number of strains in this study of M. tuberculosis and S. aureus is too small to correctly approximate the proportions, so we expect that the actual proportions will turn smaller.

This experiment showed also the good scalability of CAMBer. We ran our largest case study on 41 *E. coli* strains on a cluster of 17 computer nodes using Sun Grid Engine technology to spread the computations. Most time consuming were blast computations, which took around two days. We also found that it took around 9 hours to compute the closure and the consolidation graph assuming precomputed blasts. In the *E. coli* case study computations of the refinement took around 2 hour. We also ran the *S. aureus* case study on the same cluster. It took around 4 hours to compute the closure assuming precomputed blasts, and around 1 hour to compute the refinement. However, we did the case study on *M. tuberculosis*—which is much smaller—using a single computer with 16 cores, 3000 MHz, 64 GB RAM. It took about 10 hours to compute the consolidation graph (including time consuming blast computations) and only several minutes to perform the refinement procedure.

All the above statistics for the computational experiments suggest that CAMBer may be a useful utility in comparing and revising annotations of closely related bacterial genomes.

Input data, software used in the paper (written in Python), and detailed xls files with results of the case study experiments are available at http://bioputer.mimuw.edu.pl/camber.

## Competing interests

The authors declare that they have no competing interests.

## Authors contributions

All authors contributed to design of the method, analysis of results and writing of the manuscript. MW wrote software and performed experiments. All authors read and approved the final manuscript.

## Supplementary Material

Additional file 1 **summary of the M. tuberculosis case study results** An Excel file containing the summary of CAMBer results (after refinement) for the *M. tuberculosis* case study. Click here for file

Additional file 2 **summary of the S. aureus case study results** An Excel file containing the summary of CAMBer results (after refinement) for the *S. aureus* case study. Click here for file

Additional file 3 **summary of the E. coli case study results** An Excel file containing the summary of CAMBer results (after refinement) for the *E. coli* case study. Click here for file
